# Time series prediction with improved neuro-endocrine model

**DOI:** 10.1007/s00521-013-1373-3

**Published:** 2013-03-17

**Authors:** Debao Chen, Jiangtao Wang, Feng Zou, Wujie Yuan, Weibo Hou

**Affiliations:** 1 The School of Physics and Electronic Information, Huai Bei Normal University, Huaibei, 235000 China; 2 The School of Mathematical Science, Huai Bei Normal University, Huaibei, 235000 China

**Keywords:** Neuro-endocrine model, Neural network, Particle swarm optimization (PSO), Time series prediction

## Abstract

The paper is focused on improving the performance of neuro-endocrine models with considering the interaction of glands. Comparing to conventional neuro-endocrine models, the concentration of hormone of one gland is modulated by those of others, and the weights of cells are modulated by the improved endocrine system. The interacted equation among all glands is designed and the parameters of them are chosen with theory analysis. Because all the parameters of the model are constants when the system reaches the equilibrium state, particle swarm optimization algorithm is utilized to search the optimal parameters of the model. The theory analysis indicates that the performance of neuro-endocrine model is better than or at least equal to that of corresponding artificial neural network. To indicate the effectiveness of the proposed model, some time series from different research fields, which are used in some literatures, are tested with the proposed model, the results indicate that the proposed model has some good performance.

## Introduction

A time series is a sequence of regularly sampled quantities out of an observed system, a reliable time series prediction method can help researchers model the system and forecast its behaviors [1]. In recent years, many prediction methods have been proposed to solve time series prediction problems. Among those methods, artificial neural networks (ANNs) have played a very important role since they can model both nonlinear and linear time series. The reviews of ANNs for time series prediction before 2006 are introduced in [2], and some other methods are added in this paper. Different recurrent neural networks are presented for time series prediction in [3]. Radial basis function (RBF) neural networks are utilized for time series prediction in [4–6]. To improve the global performance of neural network, recently, neuron models with simple structure and lower computational complexity are proposed for time series prediction [7, 8], and some efficient results are derived. In addition, simulating with interaction between neural system and endocrine system in biology, neuro-endocrine model is proposed recently to improve the performance of artificial neural network. Neuro-endocrine model in terms of biological inspiration is developed for simple seeking problem [9], and the ideas of glands by introducing a “pool and release” mechanism for the glands are extended in [10]. Several potential advantages of a neuro-endocrine controller over other modulation techniques intended for ANNs are introduced in [11]. Neural, immune, and endocrine systems are introduced and the method of how to modify weights of neural network by hormones is described though the testing example is not given [12]. An artificial neuro-endocrine kinematics network is designed to aid avoiding obstacle in legged robot [13], and an adaptive artificial neural-endocrine (AANE) system is proposed to help robotic leaning online and exploiting environmental data according to sensor data and actions [14]. Many applications of neural-endocrine model are almost centered in robotic fields, and there are few applications of neural-endocrine model for time series prediction so far. In addition, the engineering model of interaction between different glands is not been formed though the phenomenon is common exist in biology. The main motivation of the paper is to study the interaction mechanism of different glands and how the neural network is regulated by the improved neuro-endocrine model. Moreover, how to improve the predictive accuracy of time series is also studied.

The rest of the paper is arranged as follows. The basic concept of time series prediction is described in Sect. 2. In Sect. 3, the improved neuro-endocrine model based on feed-forward neural network with considering the interactions of different glands is introduced. LDWPSO for the improved neuro-endocrine model is introduced in Sect. 4. In Sect. 5, some applications and results are introduced. Some conclusions and future works are described in Sect. 6.

## Time series prediction

A time series is a sequence of vectors, *x*(*t*), *t* = 0,1,…, where *t* represents elapsed time. In general, *x* might be a value which varies continuously with time *t*. In practice, *x* will be a sample of discrete data points, equally spaced in time, for a given physical system with a fixed sampling rate. The sampling rate at which samples are taken dictates the maximum resolution of the model, but it is not always the case that model with the highest resolution has the best predictive power [15].

Time series prediction by neural network is to forecast future developments of the time series from value of *x* in the current time or before. It can be described as finding a appropriate function $f:R^{N} \to R$ to obtain an estimate of *x* at time *t* + *k* from the *N* time steps back from time *t*. It can be described as follows.

1 $$x(t + k) = f(x(t),x(t - 1), \ldots ,x(t - N + 1) $$

Because time series prediction problem is complex, it is often used to check the effectiveness of intelligent algorithm.

## The improved neuro-endocrine model (INEM)

**Fig. 1:**
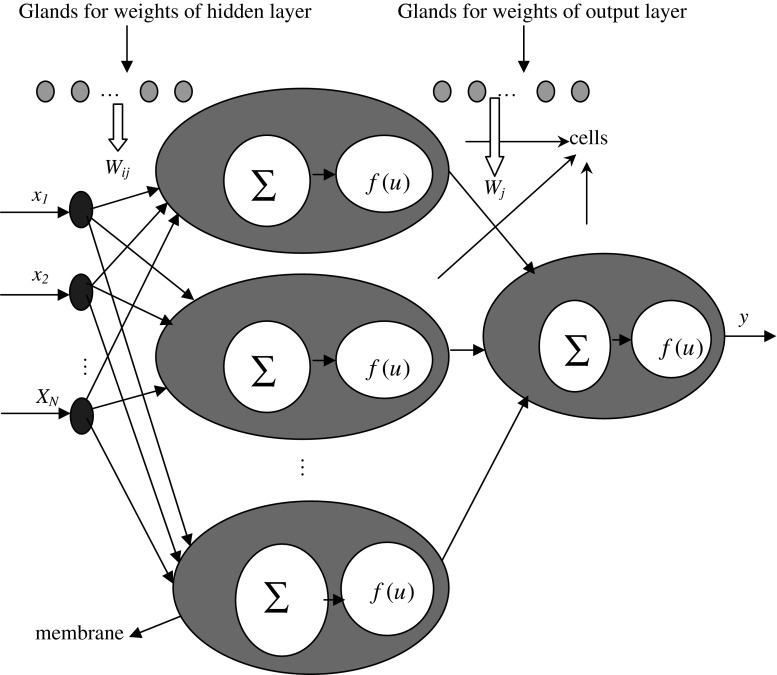
Neuro-endocrine model based on feed-forward neural network without interaction of glands

In the neuro-endocrine model, the outputs of cells are caused by outside stimuli. Neural cells express receptors for cytokines, hormones, and neurotransmitters. The endocrine system’s function is to secrete hormones into the blood and other body fluids, with the aim to regulate the behavior of neurons. There are a large number of components that make up the system, including glands such as the thyroid, the pineal, and the thymus. Hormones provide feedback to the brain affect neural processing. The neruo-endocrine model, without interaction between glands, based on feed-forward neural network is shown in Fig. 1.

Figure 1 shows that the model is based on the traditional feed-forward neural network, and the glands are responsible for producing the hormones according to certain stimuli. These hormones then modulate the behavior of the neural network by modifying its weights. Each cell has a sensitivity and a match to each hormone, the output of cell is shown in Eqs. (2), (3) and (4).

2 $$u = \sum\limits_{i = 1}^{{n_{x} }} {w_{i} x_{i} \prod\limits_{j = 1}^{{n_{g} }} {C_{j} S_{ij} M_{ij} } } - b $$

3 $$M_{ij} = \frac{1}{1 + dis(i,j)} $$

4 $$f_{o} = \frac{1}{{1 + e^{ - u} }} $$

where, *x*_*i*_ is the input for the cell, *w*_*i*_ is the weight of *i*th input for the cell, *n*_*x*_ is the number of inputs, *n*_*g*_ is the number of glands in the system, *C*_*j*_ is the concentration of hormone of *j*th gland, *S*_*ij*_ is the sensitivity of the connection of receptor *i* to hormone *j*, *M*_*ij*_ is the match between the receptor *i* and hormone *j* which is defined in Eq. (3), *dis* is the distance measure function. *b* is the threshold of the cell. For a model with *N* cell in hidden layer and one cell in output layer, there are *n*_*g*_ glands for hidden layer and *n*_*o*_ glands for output layer. It is obviously in Eq. (2), the interaction (which common existed in biology) between glands is not considered.

**Fig. 2:**
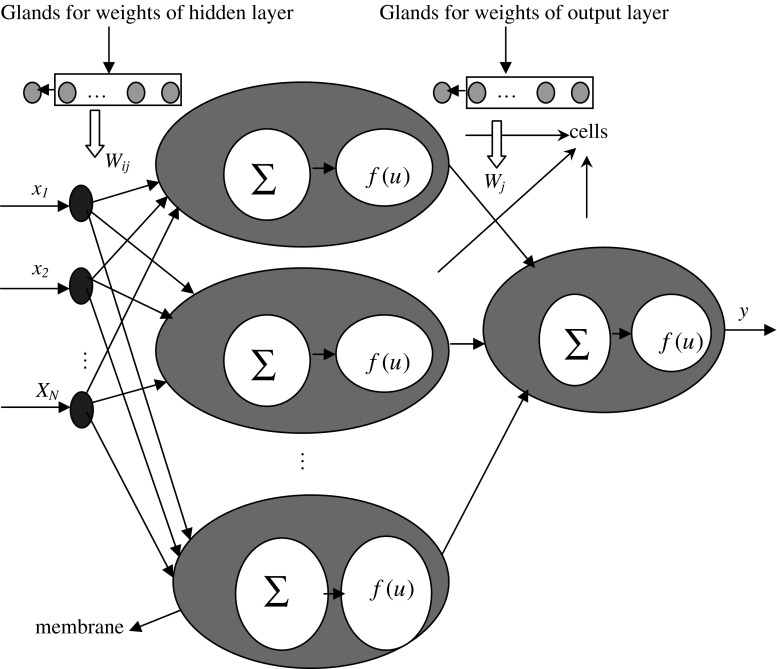
Neuro-endocrine model based on feed-forward neural network with interaction of glands

Considering the interaction between different glands, an improved neruo-endocrine model with feed-forward neural network is presented. The structure of the model is shown in Fig. 2.

Figure 2 shows that concentration of hormone of one gland is modulated by those of others, and the next task is to build an appropriate equation to represent the interactions of all glands. The basic principle is that if a gland releases more hormone it will affect the hormones of other glands in large degree. The interaction coefficient of *i*th gland caused by other glands can be shown in Eqs. (5) and (6).

5 $$AF_{i} = \frac{K}{{1 + e^{{ - MO_{i} }} }} $$

6 $$MO_{i} = \prod\limits_{h = 1,h \ne i}^{{n_{g} }} {C_{h} } $$

where, *AF*_*i*_ is the interaction coefficient of *i*th gland, *C*_*h*_ is the concentration of hormone of *h*th gland. In general, the interaction coefficient is less than or equal to one. In addition, Eq. (2) shows that if the parameters such as *C*_*j*_ *, S*_*ij*_ *, M*_*ij*_ are equal to one, the performance of neuro-endocrine model as shown in Fig. 1 will be the same as general feed-forward neural network. This might be explained as that the neuro-endocrine model at least has the same performance as general feed-forward neural network; if the parameter is appropriate, the performance of neuro-endocrine model might better than that of general feed-forward neural network. To ensure the basic performance of the improved model, *K* is chosen as shown in Eq. (7).

7 $$K = \frac{1.0}{{1 + e^{( - 1)} }} = 0.7311 $$

With this analysis, the outputs of the cells in Fig. 2 are displayed in Eqs. (8) and (9).

8 $$u = \sum\limits_{i = 1}^{{n_{x} }} {w_{i} x_{i} \prod\limits_{j = 1}^{{n_{g} }} {C_{j} S_{ij} M_{ij} } } AF_{j} - b $$

9 $$f_{o} = \frac{1}{{1 + e^{ - u} }} $$

The parameters in Eqs. (8) and (9) are the same as in Eqs. (2), (3), (4), and (5). The Eqs. (5) and (6) are fitted for cells in hidden layer and output layer. In this model, the interaction of other glands for *j*th gland is determined by the multiply of concentration of hormone of other glands. For a model with *N* cell in hidden layer and one cell in output layer, there are *n*_*g*_ glands for hidden layer and *n*_*o*_ glands for output layer, the number of parameters is the same as it in Fig. 1. For the operator in the improved model is more complex than the model of Fig. 1, the computation cost in one iteration is large than that in Fig. 1, but if the prediction accuracy or the convergent velocity is better than the models in Fig. 1, the improved model will be an efficient method for time series prediction. The number of glands for cells in hidden layer and output layer is determined by trail and error method. Firstly, the number of glands is one, then it will be increased gradually till the accuracy of the system is not changed obviously.

## LDWPSO for the improved neuro-endocrine model

### LDWPSO algorithm

PSO is an evolutionary algorithm paradigm which imitates the movement of birds or fish schooling looking for food. It is reported by Kennedy and Eberhart in 1995 [16]. In the method, each particle has a position variable (*P*_*i*_) and a velocity variable (*V*_*i*_). Each particle adjusts its position and velocity according to the best position in current generation (*gbest*) and the position which it has been achieved so far (*pbesti*). The updating equations of the velocity and position of the particles are displayed as follows:

10 $$V_{i} (t + 1) = wV_{i} (t) + c_{1} r_{1} (\varvec{P}_{pbesti} (t) - \varvec{P}_{i} (t)) + c_{2} r_{2} (\varvec{P}_{gbest} (t) - \varvec{P}_{i} (t)) $$

11 $$\varvec{P}_{i} (t + 1) = \varvec{P}_{i} (t) + V_{i} (t + 1) $$

**Fig. 3:**
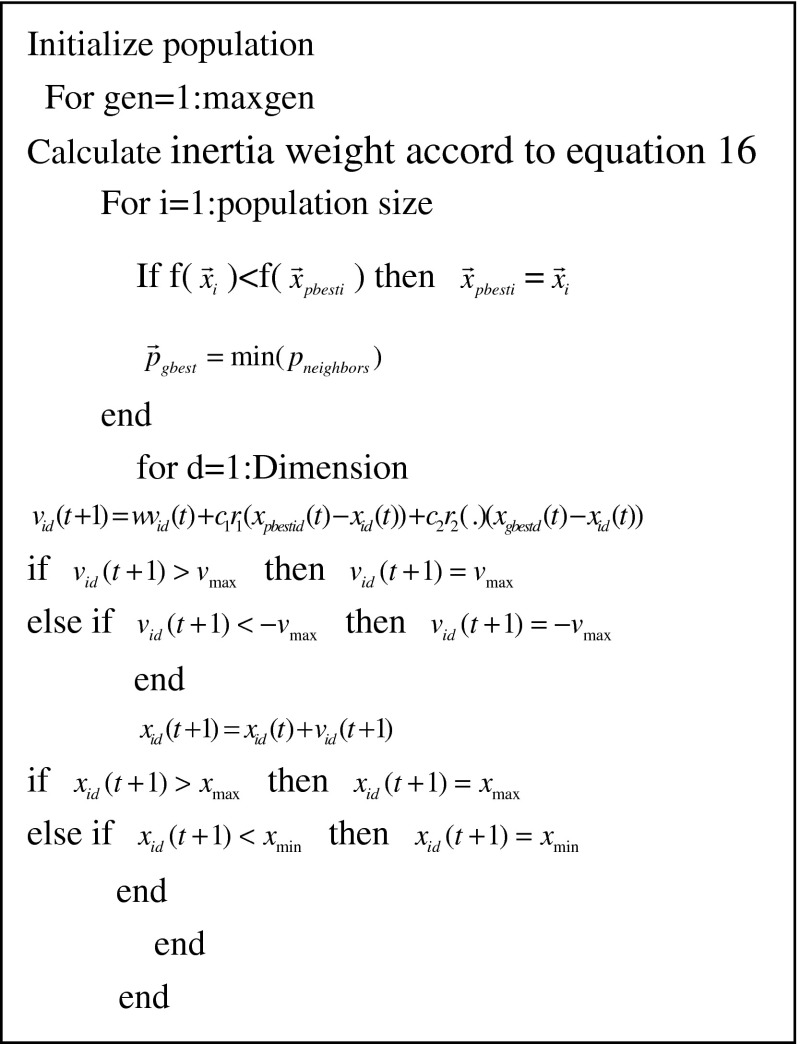
Pseudocode of LDWPSO algorithm

In Eqs. (10) and (11), *c*_*1*_ and *c*_*2*_ are often set to be constant value 2, *r*_*1*_ and *r*_*2*_ are two random uniformly distributed values in domain [0,1]. *w* is inertia weight, large inertial weight benefits for global search, a small one facilitates local search. To improve the performance of standard PSO, inertia weight decreasing linearly from a relative large value to a small one is used [17]. It can be shown in Eq. (12).

12 $$w = w_{\hbox{max} } - gen*\frac{{w_{\hbox{max} } - w_{\hbox{min} } }}{{gen_{\hbox{max} } }} $$

where, *w*_max_ = 0.9, *w*_min_ = 0.4 are the maximum and minimum values of inertia weight, respectively. *gen* is the current generation, *gen*_max_ is the maximum evolutionary generation. The initial value of *w* is relative large. The swarm has good global search ability in the beginning and has good local search ability at the end of evolution. Equations (10) and (11) show that the new positions of particles are determined by the best solutions (*gbest*) of current generation and the best positions (*pbesti*) which the particles have been achieved so far. The pseudocode of LDWPSO algorithm is shown in Fig. 3.

Where, $v_{ \hbox{max} }$ is the allowable maximum velocity of particles, $P_{ \hbox{max} } ,P_{ \hbox{min} }$ are the high and low bounds of positions.

### Optimizing the parameters of the improved model neuro-endocrine model with LDWPSO

1. Parameters representation

**Fig. 4:**
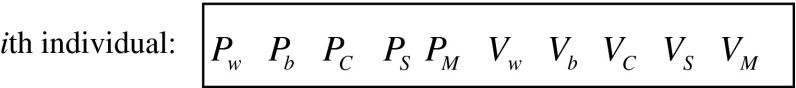
The representation of parameters for *i*th individual

The representation of parameters for *i*th individual is displayed in Fig. 4. *P* with subscript suffix is the position of individual and *V* with subscript suffix is the velocity of individual.

2. The steps of algorithm

The basic steps of the algorithm are shown as follows.

*Step 1*. Set initial parameters *c*_*1*_ = *c*_*2*_ = 2, the maximum and minimum values of inertia weight *w*_max_ = 0.9, *w*_min_ = 0.4, the maximum evolutionary generation *gen*_max_, the allowable maximum velocity $v_{ \hbox{max} }$, allowable maximum position and minimum position $P_{ \hbox{max} } ,P_{ \hbox{min} }$.

*Step 2*. Initialize the positions and velocities of the particles randomly according to the structure of Fig. 4.

*Step 3*. Execute the operators as follows.

1. Calculates inertia weight in current generation according to Eq. (12).
2. Calculates the outputs of the models according to Eqs. (8) and (9).
3. Calculates the mean squared error (MSE) between the real samples and outputs of model of each particle according to Eq. (13)
  13 $${\text{MSE}} = \frac{1}{{N_{\text{sample}} }}\sum\limits_{i = 1}^{{N_{\text{sample}} }} {(O_{s} (i) - y_{s} (i))^{2} } $$
  where, MSE(*i*) is the mean squared error function, $N_{\text{sample}}$ is the number of samples, $O_{s} (i)$ and $y_{s} (i)$ are the real output and output of current models.
4. Calculates the fitness value of all particles according to Eq. (13).
  14 $$fit(i) = \frac{1.0}{\text{MSE}} $$
  In Eq. (14), *fit*(*i*) is fitness value of *i*th particle.
5. Calculates the best position $P_{gbest}$ and the best position which the particle has been achieved so far.
6. Modify the position of all particles according to Eqs. (10) and (11), all the position and velocity should abide the follow rules.
  15 $${\text{if}}\;v_{i} (t + 1) > v_{\hbox{max} } \; {\text{then}}\;v_{i} (t + 1) = v_{\hbox{max} } ,{\text{ else if}}\;v_{i} (t + 1) < - v_{\hbox{max} } \;{\text{then}}\;v_{i} (t + 1) = - v_{\hbox{max} } $$
  16 $${\text{if}}\;x_{i} (t + 1) > x_{\hbox{max} } \; {\text{then}}\;x_{i} (t + 1) = x_{\hbox{max} } ,{\text{ else if}}\;x_{i} (t + 1) < x_{\hbox{min} } \; {\text{then}}\;x_{i} (t + 1) = x_{\hbox{min} } $$
7. If the maximum generation does not arrive, go to (1), else the evolutionary processing is ended.

*Step 4*. Compare the optimal model and real model according to testing samples.

## Applications and results

### Experiments setting

To test the effectiveness of the proposed models, 5 time series come from different research fields are utilized to evaluate the methods, and these series are used in some other papers to evaluate the artificial model. These time series are named Mackey–Glass (MG) [18], Box–Jenkins (BJ) [12], Electroencephalogram (EEG) data [8], IBM common stock closing prices [19], and Canadian Lynx data [20]. Neural network model and neuro-endocrine model without interaction of glands are also simulated, and the results of some existed model are cited to compare to the improved model. The training parameters of the models are set as follows.

The maximum training generation is 5000, *c*_*1*_ = *c*_*2*_ = 2, *w*_max_ = 0.9, *w*_min_ = 0.5, $P_{ \hbox{max} } = 30, P_{ \hbox{min} } = - 30$, the population size is 20. The number of glands for cells in hidden layer is 3 and it is 2 for cells in output layer . The other parameters of the five series are given in their simulation experiments. All the data sets are normalized between 0.1 and 0.9. The initial positions and velocities are generated randomly between 0 and 30. All the experiments are simulated 30 runs with Matlab 7.1 on Pentium VI computer.

### Mackey–glass time series (MG)

**Fig. 5:**
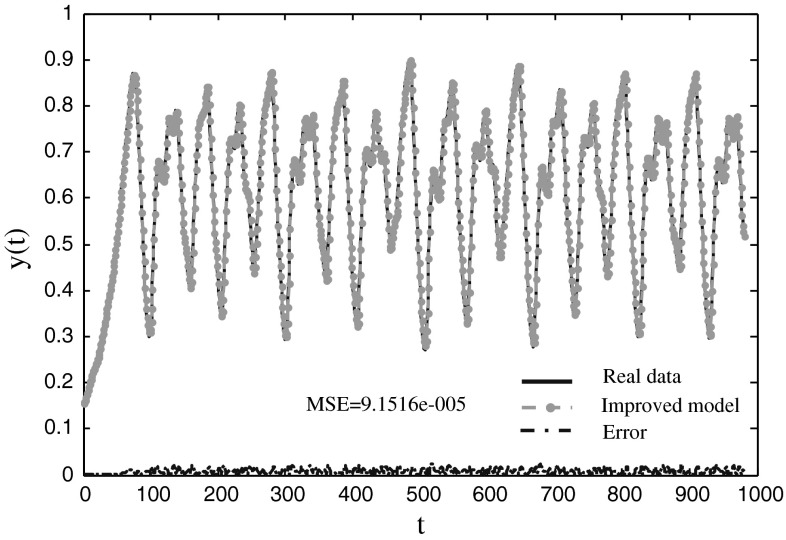
The prediction results of the MG time series using the improved model

The chaotic Mackey–Glass differential delay equation is recognized as a benchmark problem that has been used and reported by a number of researchers for comparing the learning and generalization ability of different models. The series is a chaotic time series generated from the following time-delay ordinary differential equation.

17 $$\frac{dy(t)}{dt} = \frac{ay(t - \tau )}{{1 + y^{10} (t - \tau )}} - by(t) $$

where, *τ* = 17, *a* = 0.2, and *b* = 0.1. The goal of this model is using the earlier points *y*(*t*), *y*(*t*−6), *y*(*t*−12), *y*(*t*−18) to predict *y*(*t* + 1). The training is performed on 480 samples, and the 500 samples are used for testing the generalization ability of the model. The number of cells in hidden layer is 3. This problem is often adopted as a benchmark to evaluate the performance of artificial model [21–24]. The best, the average, and the standard deviations of MSEs for training and testing are shown in Table 1, the average convergent times of CPU with a given threshold within the bracket are also displayed in the table. RMSE is usually used to compare the performance of intelligent models in some literatures, and it is also used in this paper for comparing the performance. The comparison results of the prediction error of different models are shown in Table 2. The prediction results of the improved model of training and testing are displayed in Fig. 5.

**Table 1:** The training and testing performance for predicting the MG time series with three models

	Neural model	Neuro-endocrine model without interaction of glands	Improved model
Training			
Mean	0.9122	2.0758e-004	**8.4494e-005**
Std	1.4680	1.3380e-004	**3.0569e-005**
Best	5.4702e-005	5.2127e-005	**4.0670e-005**
CPU time (0.001)		42.7660s	**36.9530s**
Successful ratio	83.3 %	100 %	100 %
Testing			
Mean	1.0366	2.1038e-004	**8.6815e-005**
Std	1.6627	1.4536e-004	**3.3291e-005**
Best	5.2078e-005	5.5016e-005	**4.1827e-005**

Bold values indicate the best results

**Table 2:** Comparison results of the prediction error of different models for Mackey–Glass Time Series

Methods	RMSE
Auto-regressive model [31]	0.19
Cascade correlation NN [31]	0.06
Sixth-order polynomial	0.04
Linear predication method	0.55
Wang and Mendel [23] Product T-norm	0.0907
GA and Fuzzy system [21]	0.049
PG-RBF network [22]	**0.0028**
WNN + hybrid [31]	0.0059
Feed-forward neural model with PSO	1.0181
Neuro-endocrine model without interaction of glands	0.0145
Improved model	0.0093

Bold value indicates the best result

Table 1 shows that the mean MSEs for training and testing data of the improved neuro-endocrine model are better than those of the other two methods. The two neuro-endocrine models are all converged to the optimal solution, and the mean time of CPU with ANN cannot be given because the successful ratio of ANN is 83.3 %. The mean time of CPU with the improved method is less than neuro-endocrine model without interaction of glands when the threshold of solution is 0.001. The standard deviation of the improved method is the smallest of the three models. Table 2 displays that the RMSE of the improved model is almost better than the other models except that it of PG-RBF network [22] and WNN with hybrid models [24]. Figure 5 shows the improved model follows the dynamic behavior with small deviations.

### Box–Jenkins gas furnace time series (BJ)

**Fig. 6:**
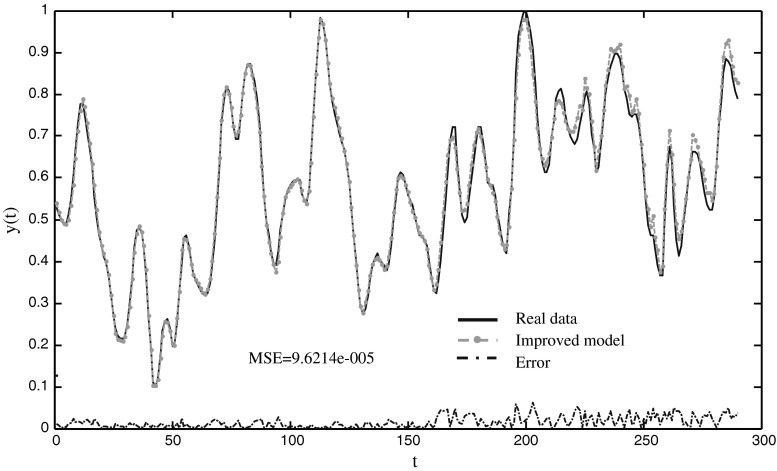
The prediction results of the BJ time series using the improved model

The Box–Jenkins gas furnace data set was recorded from a combustion process of a methane–air mixture [8]. There are 296 pairs data *y*(*t*), *u*(*t*), from *t* = 1 to *t* = 296. *y*(*t*) is the output CO_2_ concentration and *u*(*t*) is the input gas flowing rate. To test the performance of the improved model for high dimension system, *u*(*t*−1), *u*(*t*−2),…, *u*(*t*−6), *y*(*t−*1), *y*(*t−*2), *y*(*t−*3), *y*(*t−*4) are utilized to predict *y*(*t*). The training is performed on 148 samples and the model is tested on 150 samples. The number of cells in hidden layer is 4. The best, the average, and the standard deviations of MSEs for training and testing are shown in Table 3, and the CPU time and the successful ratio of the models are also given in it. Some comparison results of the prediction error of different models are shown in Table 4 [25–32]. The prediction results of the improved model for BJ datasheet are shown in Fig. 6.

**Table 3:** The training and testing performance for predicting the Box–Jenkins gas furnace dataset with three models

	Neural model	Neuro-endocrine model without interaction of glands	Improved model
Training			
Mean	2.0565e-004	2.4100e-004	**1.4412e-004**
Std	7.0618e-005	**1.1757e-006**	2.7320e-005
Best	1.0985e-004	1.0721e-004	**8.6327e-005**
CPU time (0.001)	**12.1710s**	30.2618s	38.2810s
Successful ratio	100 %	100 %	100 %
Testing			
Mean	2.3148e-004	2.6012e-004	**1.6027e-004**
Std	7.2019e-005	**1.3281e-006**	3.0143e-005
Best	1.2311e-004	1.1694e-004	**9.0217e-005**

Bold values indicate the best results

**Table 4:** Comparison results of the prediction error of different models for Box–Jenkins gas furnace dataset

Method name	Inputs	RMSE
ARMA [26]	5	0.843
Tong’s model [29]	2	0.685
Pedrycz’s model [30]	2	0.566
Xu’s model [25]	2	0.573
Surmann’s model [27]	2	0.400
Lee’s model [32]	2	0.638
ANFIS model [28]	2	0.085
Neural tree model [31]	2	0.026
WNN + hybrid [31]	2	0.081
Feed-forward neural model with PSO	10	0.0152
Neuro-endocrine model without interaction of glands	10	0.0161
Improved model	10	**0.0127**

Bold value indicates the best result

Table 3 displays that the best and the mean MSEs of the improved model for training and testing samples are smaller than those of the other two models, and the MSE of neuro-endocrine model without interaction of glands is a little better than ANN model. The standard deviation of neuro-endocrine model without interaction of glands is smaller than the other two models, and the improved method has the largest standard deviation. The CPU cost of the improved model is longer than the other two models under condition that the threshold of solution is 0.001, and the time cost of ANN is the smallest. The table also shows that the successful ratios of all models are 100 %. Table 4 shows that the improved model has the smallest RMSE, but the number of inputs is larger then some other models. The larger number of inputs might increase the computation cost of training, but the convergent accuracy is improved. Figure 6 indicates that the testing error of the model is larger than the training model.

### Electroencephalogram (EEG) data

**Fig. 7:**
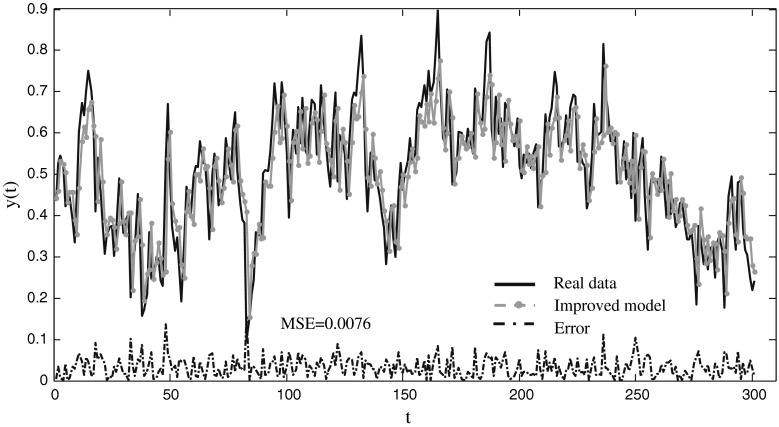
The prediction results of the EEG time series using the improved model

Electroencephalogram (EEG) data utilized in this paper was taken from http://www.cs.colostate.edu. It was recorded by Aak Keirn at Purdue university in the Electrical Engineering Department at Purdue. This problem is intentionally selected in the paper since it is observed that it cannot be predicted by linear models, and it is also used to test the effectiveness of intelligent model [8]. The goal of the model is using *y*(*t−*1), *y*(*t−*2), *y*(*t−*4), and *y*(*t−*8) to predict *y*(*t*). 150 samples are used as training data, and the other 159 data are chosen as testing samples. The number of cells in hidden layer is 2. The MSEs of the best, the average, and the standard deviations are displayed in Table 5, and the CPU time and the successful ratio of the models are also included. Comparison results of the prediction error of different models are shown in Table 6. The prediction results of the improved model for EEG are shown in Fig. 7 with MSE is 0.0076.

**Table 5:** The training and testing performance for predicting the EEG dataset with three models

	Neural model	Neuro-endocrine model without interaction of glands	Improved model
Training			
Mean	0.0078	**0.0076**	**0.0076**
Std	4.6530e-004	1.1524e-004	**1.0648e-004**
Best	0.0076	0.0075	**0.0074**
CPU time (0.001)	**0.2030s**	6.8942s	8.750s
Successful ratio	100 %	100 %	100 %
Testing			
Mean	0.0076	**0.0075**	**0.0075**
Std	4.7310e-004	1.2712e-004	**1.1086e-004**
Best	0.0075	0.0074	**0.0070**

Bold values indicate the best results

**Table 6:** Comparison results of the prediction error of different models for EEG dataset

Method name	MSE
Single multiplicative neuron model with BP [8]	0.0142
Single multiplicative neuron model with PSO [8]	0.0080
Single multiplicative neuron model with GA [8]	0.0081
Single multiplicative neuron model with CRPSO [8]	0.0081
Feed-forward neural model with PSO	0.0076
Neuro-endocrine model without interaction of glands	**0.0075**
Improved model	**0.0075**

Bold values indicate the best results

Table 5 displays that the best and mean MESs of training and testing samples of the improved method are a little better than those of the other two models, and the standard deviation is also the smallest among the three models. The three models can converge to the optimal solution when the successful threshold is set as 0.01. The computation cost of the improved model is larger than that of other models. Compare to the models in the table, the prediction error of the improved model is a little better than it of some other models except that it is derived by neuro-endocrine model without interaction of glands.

### IBM common stock closing prices model (IBMCSCP)

**Fig. 8:**
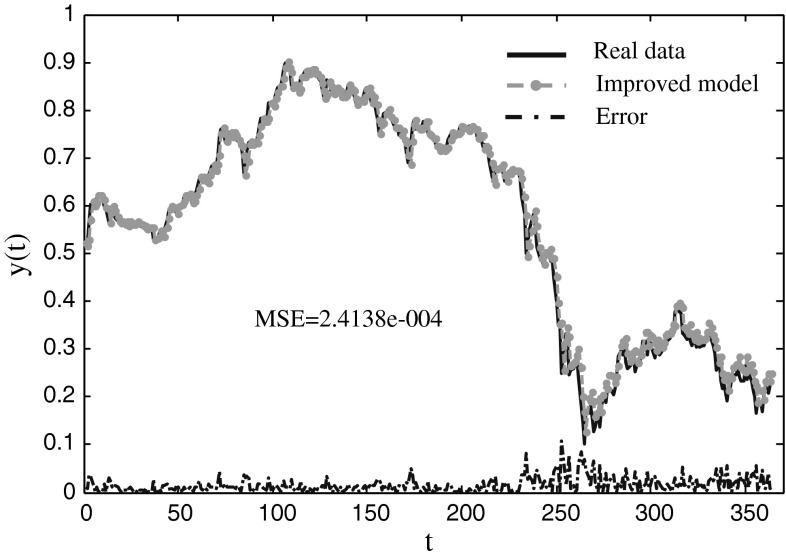
The prediction results of the IBMCSCP time series using the improved model

This time series is a real series of the daily data from May 17, 1961 to November 2, 1962. The IBM share prices show a break in the last third of the series and no obvious trend or seasonality. In the paper, *y*(*t−*1) and *u*(*t*−4) are utilized to predict *y*(*t*). 240 pair samples are chosen for training, and the other 169 samples are used for testing. Some performances of three models are displayed in Table 7, and the comparison results are shown in Table 8. Table 7 shows that the best and average MESs of the improved model are the best among the three models, and the standard deviation of it is also the smallest. The CPU time of ANN is smaller than that of other models when the threshold is 0.001, and it of the improved model is the longest. Table 8 shows that the RMES of the improved model is smaller than those of other models except SVM model. The prediction result of the improved model with MSE equal to 2.4138e-004 is shown in Fig. 8. The figure indicates that the improved model can approximate the real series in high accuracy.

**Table 7:** The training and testing performance for predicting the IBMCSCP dataset with three models

	Neural model	Neuro-endocrine model without interaction of glands	Improved model
Training			
Mean	2.4115e-004	2.4106e-004	**2.4101e-004**
Std	1.3324e-006	1.3554e-006	**1.1075e-006**
Best	2.3956e-004	2.3851e-004	**2.3820e-004**
CPU time (0.001)	**2.1034s**	4.3866s	5.2190s
Successful ratio	100 %	100 %	100 %
Testing			
Mean	2.5618e-004	2.5433e-004	**2.4712e-004**
Std	1.5541e-006	1.4718e-006	**1.0176e-006**
Best	2.4819e-004	2.4781e-004	**2.4022e-004**

Bold values indicate the best results

**Table 8:** Comparison results of the prediction error of different models for IBMCSCP dataset

Models	Inputs	RMSE
SVM [19]	4	**0.0129**
ANN [19]	6	0.0158
Feed-forward neural model with PSO	2	0.0160
Neuro-endocrine model without interaction of glands	2	0.0159
Improved model	2	0.0157

Bold value indicates the best result

### Canadian Lynx data (CLYNX)

**Fig. 9:**
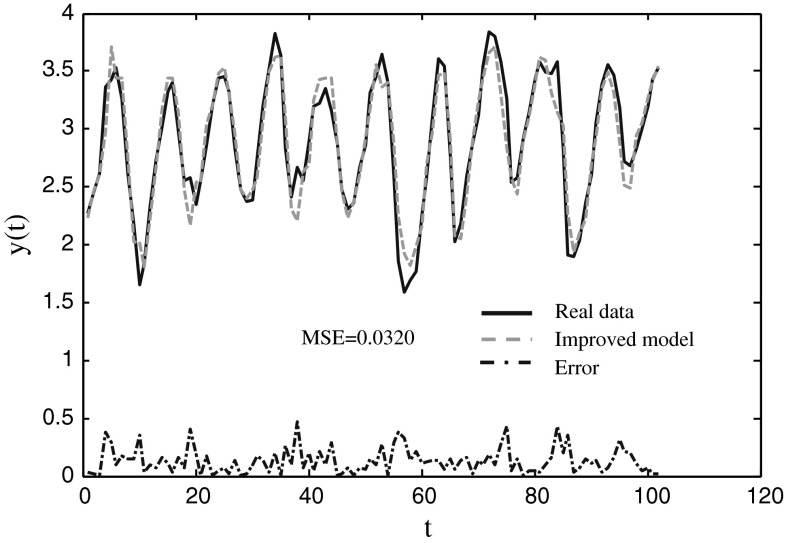
The prediction results of the Lynx time series using the improved model

This classic time series contains annual records of the numbers of Canadian lynx trapped in the MacKenzie river district of North-West Canada for the period 1821–1934 [33]. It is reported by Elton and Nicholson firstly (1942). And Moran (1953) was first to analyze the data statistically. Then it is studied by some other authors [34–36]. Following Moran [37], as well as succeeding studies and to make the series more symmetric, the original series is transformed by log^10^ first, and this method is also in this paper. Similar to some other models [38], *y*(*t−*1), *y*(*t−*2), *y*(*t−*3), *y*(*t−*4), *y*(*t−*9), *y*(*t−*11), and *y*(*t−*12) are used to predict *y*(*t)*. 100 samples from the datasheet are utilized for training, and the other 14 samples are used for testing. The number of cells in hidden layer is 3. Some performances of the three models are displayed in Table 9, and the comparison results of prediction are shown in Table 10. The actual and prediction data are shown in Fig. 9.

**Table 9:** The training and testing performance for predicting the Canadian Lynx Data dataset with three models

	Neural model	Neuro-endocrine model without interaction of glands	Improved model
Training			
Mean	0.0360	0.0357	**0.0334**
Std	0.0025	0.0022	**0.0019**
Best	0.0315	0.0316	**0.0290**
CPU time (0.04)	**30.8600s**	42.6538s	48.5780s
Successful ratio	100 %	100 %	100 %
Testing			
Mean	0.0379	0.0368	**0.0354**
Std	**0.0057**	0.0068	0.0074
Best	0.0361	0.0388	**0.0358**

Bold values indicate the best results

**Table 10:** Comparison results of the prediction error of different models for Canadian Lynx Data dataset

Models	MSE
PADD [20]	0.046
SETAR1 [34]	0.042
FAR [20]	0.036
SAR [20]	0.038
SETAR2 [38]	0.042
SBL [20]	**0.022**
GP [20]	0.028
Feed-forward neural model with PSO	0.0360
Neuro-endocrine model without interaction of glands	0.0357
Improved model	0.0334

Bold value indicates the best result

Table 9 shows that the best and the mean MESs of the improved model are smaller than those of other two models. The standard deviation of the improved model is same as that of neuro-endocrine model without interaction of glands, and it less than that of ANN model. The CPU time of the improved model is the longest among the three models. Table shows that the prediction error of the improved model is larger than the ones in SBL and GP methods, and it is less than those of other methods in the table. Figure 9 indicates that the improved model can predict the data in high accuracy.

### Comparisons using *t* test

For a thorough comparison, the *t* test [39, 40] has also been carried out. Table 11 presents the *t* values and the *P* values on every datasheets of this two-tailed test with a significance level of 0.05 between the improved model and the other two models. The number of datasheets that the improved model performs significantly better than, almost the same as, and significantly worse than the compared model is also displayed in the table. The table shows the improved model outperforms the other models for most datasheets.

**Table 11:** Comparisons between the improved method and other two models on *t* test

Datasheet			ANN	Neuro-endocrine model without interaction of glands
MG	Training	*t* value	**3.4033**	**4.9121**
		*P* value	**0.001213**	**0.000008**
	Testing	*t* value	**3.4145**	**4.5385**
		*P* value	**0.001172**	**0.000029**
BJ	Training	*t* value	**4.4509**	**19.4049**
		*P* value	**0.000039**	**0.00000**
	Testing	*t* value	**4.9958**	**18.1260**
		*P* value	**0.000006**	**0.00000**
EEG	Training	*t* value	**2.2950**	0.0000
		*P* value	**0.02537**	1.00000
	Testing	*t* value	1.1272	0.0000
		*P* value	0.2643	1.00000
IBMCSCP	Training	*t* value	0.4426	0.1565
		*P* value	0.659701	0.876182
	Testing	*t* value	**26.7136**	**22.0701**
		*P* value	**0.0000**	**0.000000**
Lynx	Training	*t* value	**4.5352**	**4.3337**
		*P* value	**0.000029**	**0.000059**
	Testing	*t* value	1.4659	0.7630
		*P* value	0.148076	0.448556
	Better		7	6
	Same		3	4
	Worse		0	0

Bold values indicate that the performance of the improved model is better than the others with *t* test

## Conclusion and future works

In this paper, the interaction between glands is designed to improve the performance of neuro-endocrine model, the interaction equation for concentration of hormone of one gland is modulated by the others is formed, and the parameter of the equation is given. With training of LDWPSO, three models is simulated, the results indicate that the accuracy of the improved model is better than the others. According no free lunch theory, the computation cost of the improved model is longer than the other two models for some datasheets. This is also the shortcoming of the model. The future works for the improved model are to design new method to decrease the computation cost and find the new method to determine the optimal number of glands in different layers.
